# Identification of novel exons and transcribed regions by chimpanzee transcriptome sequencing

**DOI:** 10.1186/gb-2010-11-7-r78

**Published:** 2010-07-23

**Authors:** Anna Wetterbom, Adam Ameur, Lars Feuk, Ulf Gyllensten, Lucia Cavelier

**Affiliations:** 1 Department of Genetics and Pathology, Rudbeck laboratory, Uppsala University, SE-751 85 Uppsala, Sweden

## Abstract

**Background:**

We profile the chimpanzee transcriptome by using deep sequencing of cDNA from brain and liver, aiming to quantify expression of known genes and to identify novel transcribed regions.

**Results:**

Using stringent criteria for transcription, we identify 12,843 expressed genes, with a majority being found in both tissues. We further identify 9,826 novel transcribed regions that are not overlapping with annotated exons, mRNAs or ESTs. Over 80% of the novel transcribed regions map within or in the vicinity of known genes, and by combining sequencing data with *de novo *splice predictions we predict several of the novel transcribed regions to be new exons or 3' UTRs. For approximately 350 novel transcribed regions, the corresponding DNA sequence is absent in the human reference genome. The presence of novel transcribed regions in five genes and in one intergenic region is further validated with RT-PCR. Finally, we describe and experimentally validate a putative novel multi-exon gene that belongs to the ATP-cassette transporter gene family. This gene does not appear to be functional in human since one exon is absent from the human genome. In addition to novel exons and UTRs, novel transcribed regions may also stem from different types of noncoding transcripts. We note that expressed repeats and introns from unspliced mRNAs are especially common in our data.

**Conclusions:**

Our results extend the chimpanzee gene catalogue with a large number of novel exons and 3' UTRs and thus support the view that mammalian gene annotations are not yet complete.

## Background

It is generally believed that comparisons at the genome and transcriptome levels are powerful strategies towards understanding the molecular differences that underlie the phenotypic divergence between humans and chimpanzees. Since the time of divergence, approximately 6 million years ago [1], the two species have acquired changes in both their genomes and their transcriptomes. The draft chimpanzee genome sequence [2] provided new opportunities to study primate biology and to understand the speciation process. Mikkelsen *et al. *[2] presented a comprehensive analysis of the chimpanzee genome and a comparative analysis with the human genome, but did not address the complexity of the transcriptome. Studies of chimpanzee transcription have been performed primarily with microarrays, covering both coding [3-8] and noncoding regions [9] of the genome. Due to the sequence divergence between the species, the use of microarrays can be problematic when arrays based on human sequence data are employed to study the chimpanzee transcriptome. To circumvent this problem, custom-made arrays with species-specific probes have been used [10,11]. Notwithstanding, all targeted expression arrays are based on *a priori *assumptions regarding the expressed parts of the genome, and are therefore not suitable for unbiased studies of transcription and discovery of novel transcripts.

Direct detection of both known and novel transcripts and exons can be achieved by complete sequencing of the cDNA population. This strategy was used by Sakate *et al. *[12], who used Sanger sequencing of cloned cDNA libraries to assemble the 5' end of 226 protein-coding chimpanzee genes and later to describe the full-length cDNAs from 87 protein-coding genes [13]. However, Sanger sequencing is not suitable for capturing the full complement of the chimpanzee transcriptome. The advent of second-generation sequencing techniques now makes it possible to directly sequence the RNA populations (RNA-Seq) to an unprecedented depth, providing information on both which genomic regions are transcribed and their expression levels. This technology has been successfully applied to eukaryotes, including yeast [14,15], *Caenorhabditis elegans *[16], mouse [17] and human [18-20]. More recently, transcriptome profiling has been employed for comparative studies of human, chimpanzee and rhesus macaque [21,22]. Blekhman *et al. *[21] used RNA-Seq to identify a large number of genes in liver, where the expression levels appear to be under natural selection in primates. They also identified a group of genes with similar expression levels between species but with a sexually dimorphic expression pattern. In another study, Babbitt *et al. *[22] used Tag-sequencing [23,24] to survey the coding and noncoding transcriptome in frontal cortex. Their results show that in addition to protein-coding genes, a group of noncoding transcripts is also conserved between the species.

Transcriptome studies usually rely on different types of annotations to determine where genes and other functional elements are located within the genome. Such gene predictions can be based either on the DNA sequence itself or on alignments of mRNAs and/or ESTs [25]. For chimpanzee, few expression data are available and gene models have therefore been based on evidence from human annotations. This results in a homocentric view that has previously made it hard to detect expression of chimpanzee-specific transcripts. However, using RNA-Seq it is now possible to measure gene expression and capture the diversity of the chimpanzee transcriptome in an unbiased way. Transcriptome sequencing of human HapMap cell lines [26] indicates that even for well-annotated genomes, it is possible to identify many novel transcripts. Pickrell *et al. *[26] describe almost a thousand novel transcribed regions (TRs) that appear to be part of existing human gene models. Many of these regions are spliced to annotated exons and most regions were putative new UTRs rather than protein-coding exons. Based on these findings we expected also to find a large number of novel TRs in the chimpanzee genome.

Here we report the results from sequencing of the chimpanzee transcriptome in brain (frontal cortex) and liver at higher coverage than previous studies [21,22]. Our samples are unique, originating from infant chimpanzees, and thus the results provide an important complement to studies of adult animals. Using the SOLiD platform, we generated over 500 million reads from the brain and liver of two chimpanzees. The sequence data were used to quantify expression of known genes and to identify novel TRs, some of which appeared to be absent from the human genome. In these analyses we assessed differences both between the tissue types and between individuals. Furthermore, by combining the novel TRs with *de novo *splice predictions we were able to detect numerous uncharacterized exons and 3' UTRs that extended existing gene models. Using this strategy we also identified a putative novel member of the ATP-cassette transporter gene family, located on chromosome 16. These novel exons and the new gene model were not included in human transcript databases, thereby suggesting that they may account for some of the uncharacterized variation between the species. In conclusion, our experimental approach enabled us to create a comprehensive catalogue of transcribed elements across tissues and individuals.

## Results

### Sample preparation, sequencing and mapping of reads

**Figure 1:**
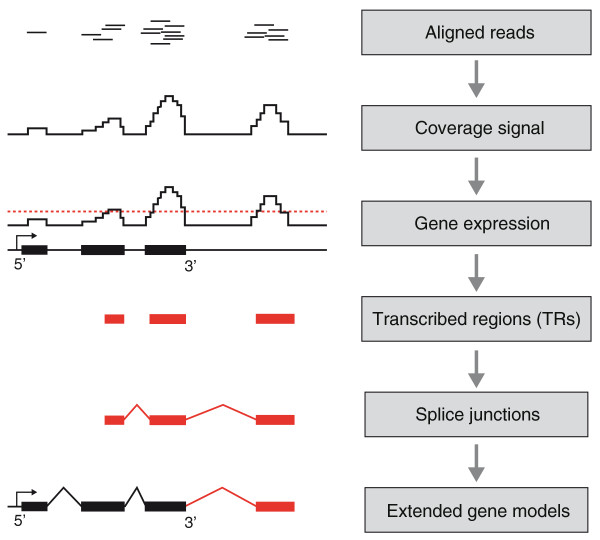
**Work flow for the bioinformatics analyses**. Sequence reads were mapped to the reference genome (PanTro2), a coverage signal was calculated across the genome and a threshold for expression was established. The threshold was initially used to determine expression of RefSeq genes and later for *de novo *detection of TRs. TRs with no previous annotations were considered to be novel and further characterized. *De novo *prediction of splice junctions was performed to join novel TRs with each other and with existing gene models.

Samples from frontal cortex and liver tissue were obtained from two young chimpanzees, one male and one female. We generated one cDNA library per tissue and individual and sequenced the fragments using the SOLiD platform. For the female chimpanzee, both 35-bp and 50-bp reads were generated (samples denoted brainF 35 bp, brainF 50 bp, liverF 35 bp and liverF 50 bp) whereas for the male only 35-bp reads were sequenced (samples denoted brainM 35 bp and liverM 35 bp). The sequencing reactions generated between 38 and 170 million reads, of which more than 40% mapped uniquely to the chimpanzee reference genome (panTro2; Table 1) when allowing for up to three mismatches for the 35-bp reads and up to four mismatches for the 50-bp reads. The subsequent analyses were performed to characterize the transcriptome repertoire, both in terms of quantifying the expression level of known genes and by identifying novel transcripts (see outline in Figure 1).

**Table 1:** Mapping summary for all six SOLiD runs

Sample	Read length	Total number of reads	Uniquely aligned reads
BrainM	35	88,598,445	34,644,708 (39.1%)
LiverM	35	78,533,657	27,872,398 (35.5%)
BrainF	35	170,016,027	79,557,661 (46.8%)
BrainF	50	38,733,951	22,897,769 (59.1%)
LiverF	35	77,388,286	27,034,253 (34.9%)
LiverF	50	58,610,173	26,393,670 (45.0%)
Total		511,880,539	218,400,459 (42.7%)

Based on the mapped reads we constructed a coverage signal profile across the chimpanzee genome. To minimize the effect of pileups of identical reads at some genomic positions, which may lead to overestimated gene expression levels, we computed the coverage signal exclusively from reads with unique starting points. This is a conservative approach and may lead to reduced dynamic range for studying gene expression. A common problem in RNA-Seq analyses is an uneven representation of the 3' and 5' ends of the mRNA [17,27]. To evaluate this aspect of the data, as well as our ability to detect known coding regions, we used the coverage signal of all chimpanzee RefSeq genes [28] and computed the average coverage for all exons and introns with different rank (for example, last exon, second to last exon and so on; Figure S1 in Additional file 1). Although the coverage signals agreed very well with the location of RefSeq exons, we observed a bias towards more reads in the 3' end of the genes. This bias was most likely due to incomplete reverse transcription of the template RNA from the oligo(dT) priming used in the first-strand cDNA synthesis.

### Quantifying gene expression and detecting transcribed regions

**Figure 2:**
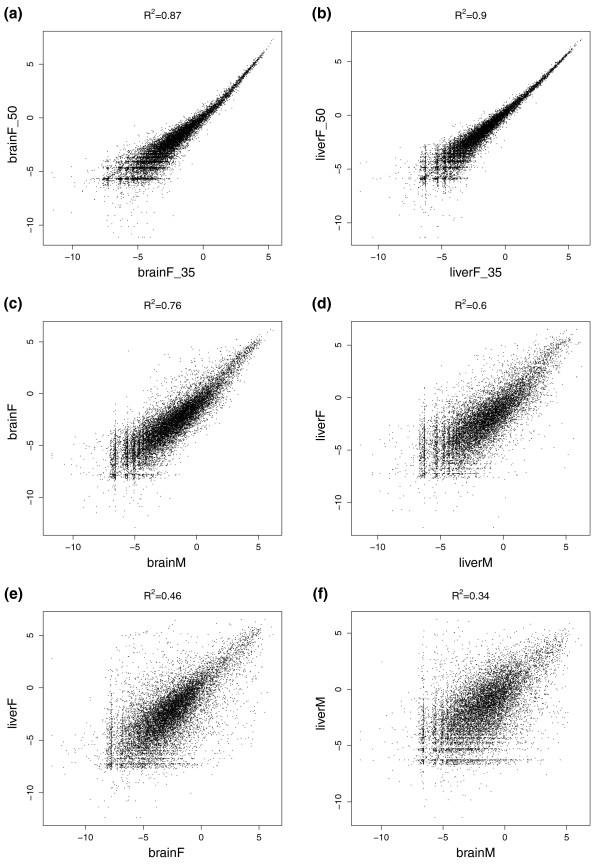
**Pearson correlations of expression signals for different sequencing runs**. **(a,b) **The correlation between 35-bp versus 50-bp reads for the datasets brainF and liverF. **(c,d) **The correlation between the two individuals in brain and liver, respectively. **(e,f) **The correlation between brain and liver within each individual. Gene expression values were estimated as the depth of coverage per million reads (dcpm), using the last 500 bp of RefSeq genes. The axes in the figures represent log2(dcpm).

To define genes, we used annotations of human and chimpanzee RefSeq genes [28], which are based on alignments of RefSeq RNAs. Gene expression was estimated using the 'average depth of coverage per million reads' (dcpm), as proposed by Hillier *et al. *[16]. Dcpm is the coverage score normalized for the total number of mapped reads. To avoid the observed 3' bias, expression was estimated only for the last 500 bp of each gene, ensuring that the expression data were comparable between genes of different lengths. Two of the samples, brainF and liverF, were sequenced with different read lengths (35 bp and 50 bp). These technical replicates showed a very high correlation of gene expression levels (Figure 2a,b), demonstrating the reproducibility of the sequencing results. Consequently, we merged the technical replicates to obtain four final datasets: brainF, liverF, brainM and liverM. A higher correlation of transcription levels was seen between identical tissues from the two individuals than between the two different tissues from the same individual (Figure 2c-f).

**Figure 3:**
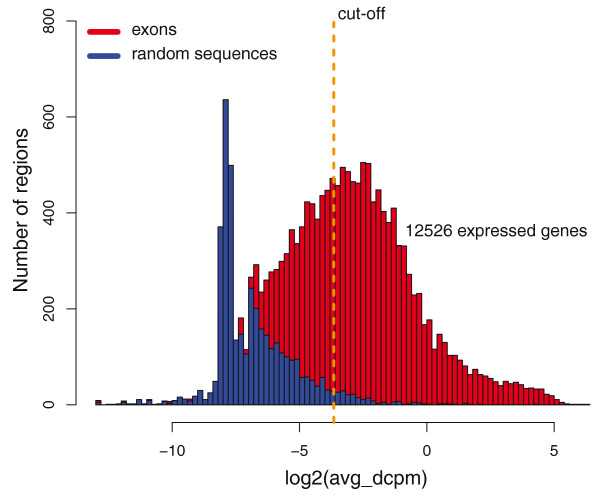
**Establishing a threshold for expression of genes and transcribed regions**. Comparison between the coverage of the last 500 bp of RefSeq exons (red) and an equal number of randomly sampled regions with the same length distribution (blue). The x-axis shows the dcpm values and the y-axis denotes the frequency of exons or random regions with a certain dcpm. Random regions do not overlap with any RefSeq exon and regions with no coverage are not shown in the distributions. The cut-off (yellow line) is placed at the top 5% in the random distribution, which is different for each sample.

To detect significantly expressed genes we defined a coverage threshold for expression. The distribution of dcpm values from the last 500 bp of all genes was compared to the coverage signal of an equal number of intergenic sequences of the same length, and on the same chromosomes, sampled at random from the chimpanzee genome (Figure 3). The two distributions had a small overlap and the cut-off was set to exclude 95% of the random sequences. We used the same approach to define a distinct threshold for each of the four samples. This resulted in a similar number of expressed genes per tissue in the two individuals, with 11,315 genes being significantly expressed in both brain samples and 8,806 genes expressed in both liver samples (Table 2).

**Table 2:** Number of expressed RefSeq genes and transcribed regions

	BrainM	BrainF	Both brains	LiverM	LiverF	Both livers
Number of expressed RefSeq genes	13,094	12,526	11,319	10,353	11,110	8,810
Total number of TRs	208,472	188,421	116,075	80,184	146,173	61,920
Number of novel TRs	79,092	76,847	19,446	17,617	47,295	6,496

The same expression level thresholds were then used for *de novo *detection of TRs across the genome, not limiting the analysis to predefined gene annotations. To reduce the frequency of false positives, we required each region to have at least 50 bp consecutively above the cut-off. Additionally, we required the regions to be expressed in the same tissue of both animals. A total of 116,075 TRs were detected in brain and 61,920 in liver (Table 2), including both regions overlapping with RefSeq genes and TRs outside of previous annotations. The coordinates for all TRs are available in Additional file 2 and can be uploaded and viewed in the UCSC Genome Browser [29,30].

### Localization and expression of transcribed regions

**Figure 4:**
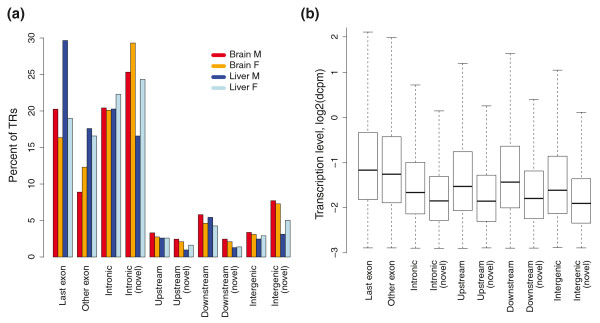
**Genomic distribution and expression level of transcribed regions**. **(a) **A histogram of the percentage of TRs in different genomic locations. The results are grouped into different genomic locations along the x-axis and the y-axis represents the percentage of TRs in each genomic location. Each tissue is plotted separately in a different color. **(b) **A boxplot of the transcription levels (measured in dcpm) for TRs in different genomic locations. The x-axis shows different genomic locations and expression values are on the y-axis. In this figure, all samples are pooled together.

Each TR was classified as exonic (overlapping a RefSeq exon), intragenic (inside a RefSeq intron), upstream (< 10 kb from the RefSeq transcription start site), downstream (< 10 kb from the RefSeq 3' end) or intergenic (all other regions) by subsequent matching to each of the categories. This way each TR may belong to only a single genomic region, although there may be overlapping gene annotations in databases. The percentage of TRs in different genomic regions is shown in Figure 4a and the absolute numbers are provided in Table S1 in Additional file 1. When considering all samples together, more than 33% of the TRs mapped to RefSeq exons and approximately one-fifth of the regions in this group were located within terminal exons. Non-exonic TRs were further compared to annotations of human and chimpanzee mRNAs and ESTs, and TRs not overlapping any of these annotations were termed 'novel'. Thus, novel TRs represent transcripts that have not previously been observed in either human or chimpanzee. The overwhelming majority of novel TRs were located in introns, followed by intergenic regions and regions in the proximity (that is, within 10 kb) of genes (summarized in Figure S2). The large accumulation of novel TRs around known genes suggests a multitude of alternative isoforms that have not been previously characterized.

The gene expression levels (measured in dcpm) for different genomic locations are shown in Figure 4b. The exonic regions had the highest dcpm and there was only a small difference in expression levels between terminal and all other exons, although more TRs were found in terminal exons counted in absolute numbers. The second highest dcpm was seen for regions within 10 kb downstream of RefSeq genes, followed by the categories 10 kb upstream, intergenic and finally intronic. Novel TRs had lower average dcpm levels than annotated TRs in the same genomic locations, thus emphasizing the potential of deep sequencing to identify TRs that have previously escaped detection due to lower transcription levels.

### Comparing expression levels in frontal cortex and liver

**Figure 5:**
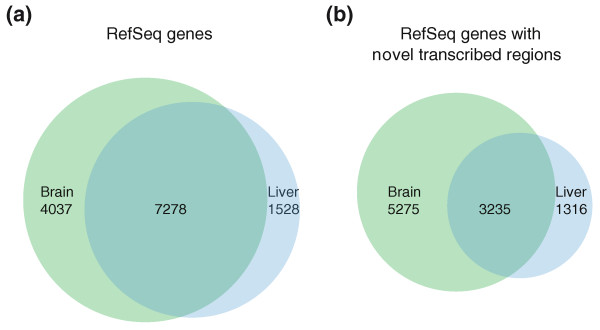
**Tissue distribution of expressed genes and genes containing novel transcribed regions**. The Venn-diagram illustrates the proportion of genes expressed only in brain, only in liver or in both tissues simultaneously. **(a) **The tissue distribution of expressed genes; **(b) **the tissue distribution of genes harboring at least one novel TR (found intronically or within 10 kb upstream or downstram of the gene).

We examined the expression of chimpanzee genes in the two tissues and were able to confirm a total of 12,843 expressed genes, with 11,315 in frontal cortex and 8,806 in liver (Figure 5a). Of these genes, 7,278 (57%) were expressed in both tissues and this group of genes had the highest average expression values (dcpm_mean _= 5.4). Liver-specific genes had slightly lower expression (dcpm_mean _= 3.9) and brain-specific genes showed even lower expression levels (dcpm_mean _= 1.5). We further examined the biological function of genes in the three categories by a Gene Ontology analysis and found that ubiquitously expressed genes were primarily involved in general biological processes such as metabolism and RNA processing (Table S2 in Additional file 1). Genes with tissue-specific expression clustered into different biological processes. Brain-specific genes were over-represented in several developmental processes, for example, neurological and anatomical structure development, and in cell adhesion. In contrast, genes with liver specific expression were over-represented in many metabolic processes, including lipid and carboxylic acid metabolism, as well as in inflammatory response (Table S2 in Additional file 1).

In addition to expression of known genes, we also identified a large number of novel TRs. The vast majority (84%) of novel TRs mapped within RefSeq introns or within 10 kb upstream or downstream of RefSeq genes. As a comparison, these extended RefSeq loci covered approximately half of the genome, and thus there was a clear enrichment of TRs in these regions. The tissue distribution of genes containing novel TRs is displayed in Figure 5b. In comparison to the results for all expressed genes (Figure 5a), a larger proportion of genes with novel TRs was found in brain than in liver. Furthermore, we noted that the overlap between tissues was lower for genes containing novel TRs than for expressed genes in general, indicating that novel TRs have a higher degree of tissue specificity. A Gene Ontology analysis showed that genes harboring novel TRs belonged to similar biological processes as was found for expressed genes in general (Table S3 in Additional file 1).

### Comparing expression levels between individuals

Although most genes were detected in both chimpanzees for each respective tissue, we observed some differences between the individuals. A total of 14,301 genes were detected in the frontal cortex samples and 80% (n = 11,319) of these were found in both chimpanzees. The corresponding figures for liver are 12,653 genes in total, with 70% (n = 8,810) shared between both individuals. Levels of gene expression were highly correlated between individuals, in both brain and liver (Figure 2c,d). We also noted that genes with individual-specific expression generally had lower expression levels than genes detected in both chimpanzees, indicating that the differences between samples is, to some extent, a result of the sequencing depth. In contrast to known genes, where a large proportion was shared between individuals, novel TRs were not shared to the same extent. Of the novel TRs, only 14% and 11% were common to both individuals in brain and liver, respectively. This reflects the fact that novel TRs were generally expressed at lower levels, and in parallel to known genes, regions with lower expression level were shared to a lesser extent between the two chimpanzees.

**Figure 6:**
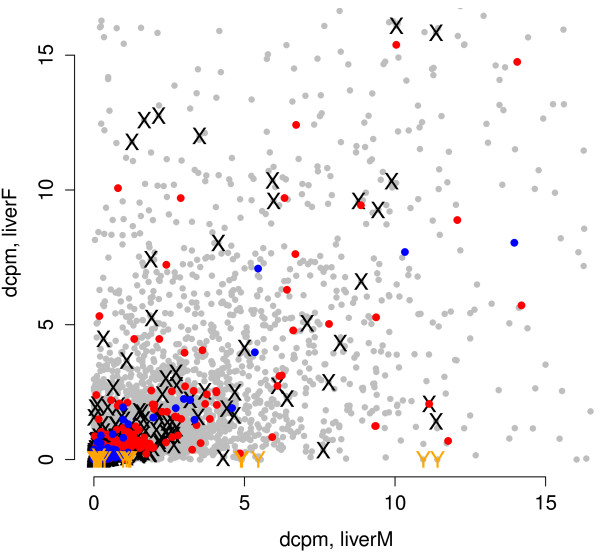
**Sexually dimorphic gene expression**. Differences in gene expression (in liver) between the two individual chimpanzees. The x-axis represents expression values (in dcpm) in the female and the male is on the y-axis; each grey dot represents the expression of one gene. Genes located on the sex chromosomes are displayed as × and Y, respectively. Overlaid are data from Blekhman *et al. *[21] with red dots indicating genes with higher expression in female than in liver, and blue dots representing the opposite scenario.

Blekhman *et al. *[21] have previously reported sexually dimorphic gene expression in liver in primates (conserved between human, chimpanzee and macaque) and to revisit this question in our data we plotted gene expression for male against female (Figure 6). As a control in our data we highlighted genes on the sex chromosomes and noted the expected pattern with genes on the Y chromosome expressed only in the male, whereas there was no clustering of × chromosomal genes. We then plotted our results for the genes reported by Blekhman *et al. *and as shown in Figure 6 we were not able to replicate the pattern of sexually dimorphic gene expression in our data. Since gene expression in primates is known to vary with age and developmental stage [7], we speculate that the observed discrepancy could reflect the different ages of the chimpanzees examined by Blekhman *et al. *and by us.

### Further characterization and validation of novel transcribed regions

We required TRs to be present in both chimpanzees and using this criterion we identified 116,075 TRs in brain and 61,920 in liver. TRs were often found in clusters, thus indicating that closely spaced TRs originated from the same transcript and with increasing sequencing depth many of the TRs are likely to merge into longer transcripts. Seventeen percent (19,446) of the TRs in brain and 10% (6,496) of those in liver did not overlap any previously annotated exons, mRNAs or ESTs. Such TRs were considered novel TRs and analyzed further to elucidate their origin and function. Current gene annotations in chimpanzee are almost exclusively based on transcriptional data originating from human and this implies that the novel TRs in our study have not been previously detected in human. The explanation for this may be either that the novel TRs are absent in human tissues or that they are expressed at low levels and have thus escaped detection.

#### Novel transcribed regions absent from the human genome

A subgroup of novel TRs could not be mapped to the human genome sequence, indicating that the DNA-sequence has been lost in human or gained in chimpanzee. Starting with the complete dataset of all novel TRs (19,446 in brain and 6,496 in liver), we selected all regions where the coordinates could not to be translated to the human genome (hg19). For these candidates, we BLASTed [31] the transcribed chimpanzee sequences to the entire human genome and selected TRs that did not give a significant match. This resulted in 285 novel TRs in brain and 77 in liver, which were not present in the human genome sequence. Such novel TRs were located both in the vicinity of known genes (that is, intronic or within 10 kb upstream or downstream) and in intergenic regions. The genomic regions that were absent in the human genome have either been lost in the human lineage or gained in the chimpanzee lineage and to deduce the evolutionary history we used the macaque genome as an outgroup. Within this subset of novel TRs approximately half (n = 133 in brain and n = 39 in liver) could not be located in either the human or the macaque genomes, thus indicating sequence gain in the chimpanzee lineage. For the remaining part of novel TRs the region was found both in the chimpanzee and macaque genomes and such regions have most likely been lost in the human lineage.

**Figure 7:**
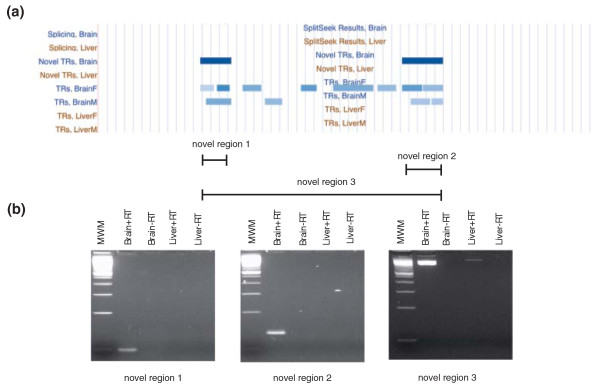
**Example of an intergenic novel transcript that was absent in the human genome**. **(a) **A view from the UCSC Genome Browser showing an intergenic region on chromosome 1. Two novel TRs are shown as blue boxes and the position of primers and the experimentally validated fragments are indicated with lines. **(b) **Results of the experimental validation with RT-PCR. The first two gels show fragments covering the two novel TRs and the third gel is a fragment spanning the junction between the novel TRs. The first lane on the gels is a 100-bp ladder (molecular weight marker, MWM), then follows the brain sample with (+RT) and without (-RT) reverse transcriptase, followed by the same set of experiments in the liver sample.

Figure 7a shows two closely located novel TRs found intergenically on chromosome 1. This region was present in both the macaque and orangutan genomes but appeared to be absent in the human genome. RT-PCR validated the two TRs as expressed and produced a transcript (approximately 800 bp) that spanned the region between the novel TRs (Figure 7b). Since this novel transcript was located in a genomic region with sparse annotation, it was difficult to predict its function. Translating the genomic sequence into amino acid code (using all six reading frames) did not reveal a long open reading frame and thus it is more likely that the two novel TRs stem from a non-coding RNA (ncRNA).

#### Novel exons and UTRs

We aimed specifically at identifying TRs that represented novel exons and UTRs. It is expected that many such TRs will extend known gene annotations and thus the TRs will be found close to known genes. This was supported by the finding that 84% of the novel TRs mapped in the vicinity of a RefSeq gene (that is, intronic or within 10 kb upstream or downstream; see Figure S2a,b in Additional file 1 for genomic distribution of novel TRs). This is an appreciable enrichment since the extended RefSeq regions covered only 52% of the genome. A proportion of these novel TRs probably represent novel exons or UTRs. Considering the sequencing bias, we expected to find mainly 3' UTRs and only to a lesser extent 5' UTRs.

Initially, we examined the length of novel TRs and found it to vary between 50 and 2,500 bp, with a median of 142 bp for brain and 151 bp for liver. The length distribution was plotted together with the lengths of RefSeq exons (Figure S3 in Additional file 1). The peak around the TR mean coincided with a similar peak in the length distribution for RefSeq exons, and the extended tail of longer novel TRs resembled the distribution of terminal exons (including annotated 3' UTRs). This comparison suggested that our collection of novel TRs was composed of a mixture of exons and 3' UTRs.

To further explore the genomic arrangement of novel TRs and to what extent they were connected to known gene annotations, we used the SplitSeek program [32] to perform a *de novo *search for splice junctions. Since the SplitSeek algorithm is not applicable for the shorter read lengths, we used only the 50-bp reads from the brainF and liverF samples in this analysis (see Materials and methods for details). A total of 1,904 splice junctions were predicted in the brainF sample and 4,279 in liverF, with as many as 90% of them mapping exactly to an exon-exon boundary in a known gene. The remaining 10% are the most interesting, since they include previously undetected splicing events connecting novel TRs to each other and to known gene models.

**Figure 8:**
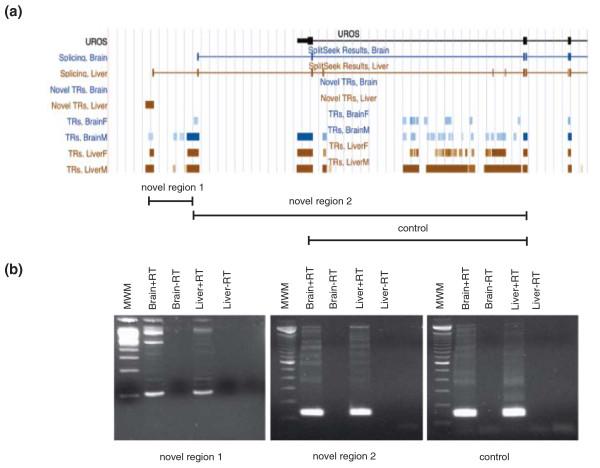
**Example of a novel 3' UTR in the *UROS *gene**. **(a) **The structure of the *UROS *gene from the UCSC Genome Browser. At the top is a schematic view of the RefSeq gene with exons as black boxes and introns as connective lines. Below are two tracks with predicted splicing events, then follows a set of tracks with detected TRs in the two tissues and at the bottom is a representation of the primers and the experimentally validated junctions between TRs. **(b) **The experimental validation with RT-PCR. The two TRs representing the novel 3' UTR are on the first two gels and a positive control of the main transcript is on the third gel. The first lane on the gels is 100 bp ladder (molecular weight marker, MWM), then follows the brain sample with (+RT) and without (-RT) reverse transcriptase, followed by the same set of experiments in the liver sample.

Having identified potential novel TRs and splice junctions, we attempted to combine these two datasets to find examples of novel exons and UTRs that were linked to a known gene model by a splice junction. We did this by intersecting the novel TRs with our predicted splice junction coordinates, and this resulted in a number of interesting candidates that we validated further with RT-PCR. Figure S4 in Additional file 1 illustrates a novel putative exon within the *KNG1 *gene. We predicted splicing from a novel exon to a neighboring downstream exon and the presence of this transcript was validated in both brain and liver samples. In addition, we validated novel exons in *MN1 *(Figure S5 in Additional file 1), *NDUFA7 *(Figure S6 in Additional file 1) and *PRDM5 *(Figure S7 in Additional file 1). We also found examples of novel 3' UTRs, as in the *UROS *gene (Figure 8) where we observed two TRs downstream of the annotated 3' UTR. One of the TRs was predicted to be novel and the other was supported by mRNA/EST data, although it was not included in the previous gene model. We were able to validate the expression of both TRs, a connection between them as well as joining the TRs to the last coding exon of the gene (without including the annotated 3' UTR). Our results demonstrated a novel 3' UTR in the *UROS *gene, which was expressed in both brain and liver.

#### Other types of novel transcripts

In addition to new exons and UTRs, the novel TRs may also stem from other types of transcripts. Poly(A)-enriched RNA is known to contain partially spliced mRNAs and this might explain a proportion of the intronic novel TRs that we observed. Furthermore, abundant transcription of different types of ncRNAs has been reported in eukaryotic genomes [33-36] and to assess this we compared our data to UCSC [29,30] annotations of 'RNA genes' and 'small nucleolar RNAs (snoRNAs)/microRNAs'. This yielded only a handful of ncRNAs (38 in brain and 23 in liver) overlapping with novel TRs. Another type of ncRNA that has been reported both in human [19] and mouse [18] is expressed repeats. Although a large proportion of primate genomes is composed of repeated sequences, these regions have not been described by previous transcriptome sequencing efforts in chimpanzee. By comparing with the RepeatMasker track from the UCSC Genome Browser [29,30] (requiring a 20% overlap), we concluded that approximately half of the novel TRs (9,193 in brain and 3,441 in liver) were derived from repeated elements. The vast majority appeared to be derived from retrotransposons such as long interspersed nuclear elements (LINEs), short interspersed nuclear elements (SINEs) and long terminal repeats (LTRs) (summarized in Table 3). Detection of expressed regions relies on mapping the sequence reads to unique positions in the genome. Thus, it is inherently harder to analyze repeated regions and the numbers in Table 3 are likely an underestimate.

**Table 3:** Percentage of novel transcribed regions that overlap with different repeat classes

	LINE	SINE	LTR	All other repeat classes
Novel TRs in brain	27%	10%	6%	4%
Novel TRs in liver	32%	10%	5%	5%

LINE, long interspersed nuclear element; LTR, long terminal repeat; SINE, short interspersed nuclear element.

#### Experimental validation of novel transcribed regions

We used reverse transcriptase PCR (RT-PCR) to verify the existence of novel TRs in seven example regions, including five genes, one intergenic region and a putative novel protein-coding gene. For novel TRs adjacent to genes, we also designed primers to amplify a fragment from the previously annotated gene, to act as a positive control and as a baseline for comparing the expression levels of novel TRs. The examples chosen for validation were selected to represent both intergenic and genic TRs. The expression levels of the validated TRs, as determined from the sequencing data, varied from just above the threshold to highly expressed regions. We were able to validate all the five novel TRs within genes, as well as the intergenic TR and for the putative novel gene we were able to validate the majority of predicted exons and junctions. The high success rate indicated that the threshold for expression was very conservative and that there exists additional RNAs that are not captured by our analyses.

In general, we observed that novel TRs had lower expression levels than the positive controls from the same gene (as judged from the intensity of the fragment on the gel). This agrees well with the results presented in Figure 4b where novel TRs have lower expression levels than annotated TRs in the same region. Furthermore, we noted that in *NDUFA7 *(Figure S6 in Additional file 1), *PRDM5 *(Figure S7 in Additional file 1) and *UROS *(Figure 8 in Additional file 1) the expression level of the control was similar in brain and liver, whereas the expression levels of the novel TRs differed between the two tissues. These results support the notion that novel TRs are tissue specific to a larger extent than transcripts in general. Some novel TRs were only detected in a single tissue, based on the threshold used to define transcription, but the region was amplified with RT-PCR in both tissues. One such example was the *KNG1 *gene, where the novel TR was initially only predicted in liver but we were able to amplify the region also in brain (although with a significantly weaker band on the gel). This further suggests that not all low abundance TRs are captured with our threshold for transcription.

### Characterization of a novel chimpanzee gene

**Figure 9:**
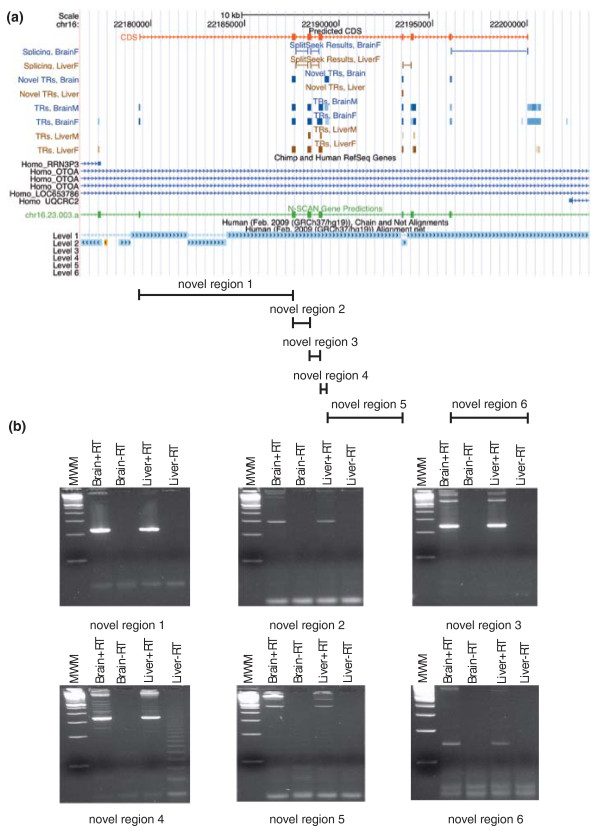
**Example of a previously uncharacterized gene**. **(a) **A view from the UCSC Genome Browser displaying the predicted novel gene, located within an intron in the *OTOA *gene. At the top is a schematic drawing of the gene with exons shown as red boxes. Then follows two tracks with predicted splice junctions and a set of tracks with TRs, shown separately for each tissue. Below are schematic models of human RefSeq genes (in blue) and an N-SCAN gene prediction (in green) in the region. At the bottom is the structure of the human genome, showing the genomic rearrangement affecting exon five in the novel gene. **(b) **The results of the experimental validation with RT-PCR. Each gel represents one exon-exon junction. The first lane on the gels from the left is a 100-bp ladder (molecular weight marker, MWM), then follows the brain sample with (+RT) and without (-RT) reverse transcriptase, followed by the same set of experiments in the liver sample. Some of the PCR products are longer than expected, indicating that some of the exons are longer than initially predicted or that there may be additional exons in the gene model.

Finally, we focused our attention on a small region on chromosome 16, located in an intron of the *OTOA *gene, which showed high enrichment of TRs and splice junctions both in frontal cortex and liver. We noted that some of the neighboring TRs were interconnected by predicted splice junction in a way that resembled the structure of a multi-exon gene. Moreover, we found that many of the TRs coincided with exons in N-SCAN gene predictions [37], and taken together this information suggested to us that protein-coding transcripts were being actively transcribed in this region. Next, we attempted to re-construct the entire gene structure based on the coordinates for TRs, predicted splice junction and N-SCAN predictions. In this way, we built a gene model consisting of eight exons and spanning approximately 20 kb. The putative gene is displayed in Figure 9. The nucleotide sequence results in an open reading frame that can be translated (in the sense direction) into a protein consisting of 328 amino acids, thus strongly suggesting that this is a protein-coding gene (see Supplementary material in Additional file 1 for DNA and protein sequences and alignments).

Searching the database of RefSeq proteins suggested that the predicted protein belonged to the ATP-binding cassette gene family. The highest similarity score was found for the mouse protein Abca15 (NP_796187.2), with 90% our predicted gene covered in the alignment and 73% identity among the aligned amino acids (see Supplementary material in Additional file 1). In addition to the eight predicted exons there was also another TR just downstream of the fourth exon. This region was expressed at significantly lower levels and inclusion of this TR disrupted the open reading frame and thus it has not been added to the proposed gene model. We used RT-PCR and validated six out of seven possible pairs of consecutive exon-exon junctions; three exon-exon junctions were also supported by predicted splice junction.

The novel gene model was further supported by EST evidence from GenBank [38,39]. We found macaque ESTs covering exons 2 to 6 in our model, and human ESTs covering exons 3, 4 and 6. We noted that the fifth exon in our gene model was skipped in the human ESTs and, as seen in the human alignment track in Figure 9, a sequence of 312 bp encompassing exon 5 has been rearranged to a different location on the same chromosome (located more than 500 kb upstream of the other exons). Removal of exon 5 in human causes a shift in the reading frame and introduces premature termination codons in the predicted protein sequence. Since the novel predicted gene appeared to be transcribed in chimpanzee and other mammals, but not in full in human, we speculate that it has become a pseudogene in human.

## Discussion

We have sequenced the chimpanzee transcriptome in frontal cortex and liver, and with more than 200 million uniquely mapping reads this is the most comprehensive dataset to date, providing a rich source for validation of existing RefSeq genes and for identification of novel TRs. Our aim was to provide a comprehensive catalogue of transcripts from protein-coding loci and to achieve this we enriched for putative coding RNAs by using oligo(dT) primers for reverse transcription. To establish a threshold for expression we compared the expression values of RefSeq genes with random intergenic regions and the threshold was set to exclude 95% of the random sequences. This approach rests on the premise that the majority of the genome is only transcribed at a low level and although this issue is still not completely resolved, a recent RNA-Seq study suggests that most of the genome is not appreciably transcribed [40]. Using this strategy we detected 11,319 expressed genes in brain and 8,810 in liver. The numbers were slightly lower than other RNA-Seq studies [21,22] of chimpanzee, indicating that our threshold was conservative. This was further suggested by the RT-PCR validation where we were able to amplify TRs with expression below the threshold. However, with the choice between finding all novel transcripts and minimizing the number of false positives, we prioritized the latter.

Having shown that we could reliably detect expression of known genes, we used the same expression threshold for *de novo *detection of TRs across the chimpanzee genome. A large number of TRs were found (over 150,000 in the two tissues) and given that many were located close together we assume that with increasing sequencing depth many TRs would merge into longer consecutive transcripts. However, to avoid introducing an arbitrary distance cut-off we did not merge nearby TRs in the analyses. TRs that were not overlapping with annotations of human or chimpanzee RefSeq exons, mRNAs or ESTs were termed 'novel', implying that the region has not previously been annotated as transcribed. Based on the oligo(dT)-priming employed for cDNA synthesis, we expected that most novel TRs would originate from polyadenylated transcripts and this was supported by the fact that 84% of the novel TRs mapped within the boundaries of RefSeq genes (that is, intronically or within ± 10 kb), thus suggesting a multitude of uncharacterized exons and UTRs. It should be noted, however, that not all exons and UTRs are necessarily protein coding. This was shown, for example, by the manual annotation of the ENCODE regions [41], which found that an appreciable proportion of transcripts from genic loci appear to be noncoding.

Novel TRs may also stem from several other types of noncoding transcripts, including expressed repeats, retained introns in unspliced mRNAs, different types of small ncRNAs or antisense transcripts. Expressed repeats and unspliced mRNAs were quite commonly observed in our data and other types of ncRNAs were not expected to be present in large numbers, considering the oligo(dT)-priming of the cDNA synthesis. Widespread transcription outside protein-coding regions in chimpanzees has been observed by Khaitovich *et al. *[9] and Babbitt *et al. *[22] and much of it appears to be conserved in primates, thus suggesting its functional importance. To address these issues in the present samples, sequencing of total RNA, with protocols providing strand-specificity, will ideally be employed.

For a small subset of approximately 350 novel TRs, the corresponding genomic sequence appeared to be absent in human. Further comparison with the macaque genome revealed that for approximately half of the novel TRs, the corresponding genomic regions had been lost in human whereas for the other half the region had been inserted into the chimpanzee genome. We have experimentally verified one such transcript where the corresponding genomic region was absent in the human genome. Since the region was also present in the macaque genome, the most plausible explanation is that the sequence was lost on the human lineage. Structural variation leading to species-specific genomic regions in both human and chimpanzee has been described by several groups, ranging from short insertions and deletions [2] to large segmental duplications [42]. If such genomic regions harbor transcriptionally active loci, they will contribute to the transcriptional diversity between the species. Thus, the group of TRs described here is intriguing and future comparative studies will have to determine whether they provide important clues to the divergent phenotypes between humans and chimpanzees.

Alternative splicing is an important mechanism for generating transcript divergence, and to assess splicing patterns in our data we used the SplitSeek software for *de novo *predictions of splice junctions. The advantage with this method is that we were able to predict exon-exon junctions that have not been described previously, in contrast to many other RNA-Seq studies that rely on methods based on predefined junction libraries. We predicted approximately 200 novel splice junctions in brain and approximately 400 in liver and this is likely to represent only a fraction of the splicing divergence. Due to the nature of our transcript data many of these novel splice junctions were found in 3' regions of genes. The difference in the number of splice junctions between brain and liver most likely reflects the higher sequence coverage in the liver sample rather than a biological difference. The expression of different transcriptional isoforms is known to vary between human tissues [20] and our analyses indicated that this was also true in chimpanzee. Tissue-specificity of noncoding transcripts has also been described previously in human [35] and our results confirmed that the same applied in the chimpanzee. Genes with novel TRs were expressed in a more tissue-specific manner than genes with no novel TRs. Furthermore, we noted several examples where the previously annotated parts of a gene were expressed at the same level in brain and liver, whereas the expression levels of novel TRs differed between the tissues. Bearing in mind that the threshold for expression was conservative, it is quite plausible that we did not capture all low abundance transcripts and thus the proportion of tissue-specific TRs may be even higher.

The samples used for sequencing originate from two infant chimpanzees and this makes the dataset unique and an important complement to previous datasets from frontal cortex [22] and liver [21] of adult individuals. Somel *et al. *[7] have previously shown that the brain transcriptome in primates is remodeled after birth and that gene expression differs with age. Although there is no parallel study of developmental changes in the liver transcriptome, it is reasonable to assume that considerable postnatal transcriptional changes occur also in the liver. The age difference is also one plausible explanation of why we were unable to replicate the sexually dimorphic gene expression observed by Blekhman *et al. *[21].

Gene annotations in chimpanzee have relied almost entirely on human mRNAs and ESTs as supporting evidence. However, not all genes are identical between the species and by using current human-centered gene annotations it is only possible to find genes where regions are present in human and absent in chimpanzee, and not the opposite. Our analyses revealed a large number of novel TRs located in the proximity of RefSeq genes and thereby extends existing annotations of as many as 9,826 genes (Figure 5b). We have a bias with higher sequencing depth towards the 3' end of transcripts, and this gives us the possibility to characterize novel 3' UTRs, even in transcripts with very low expression levels. 3' UTRs have a role in post-transcriptional regulation of gene expression, stability of the transcript and as possible targets for microRNAs. An illustrative example is the *UROS *gene (Figure 8), where we identified and validated an alternative 3' UTR that was longer than the RefSeq annotated UTR. We also identified and experimentally validated examples of putative novel exons from five genes (Figures S4, S5, S6 and S7 in Additional file 1). Such exons could possibly add novel coding regions and thereby alter the amino acid composition of the resulting protein. Similar results, with a large number of uncharacterized exons and UTRs, have been reported in human [26] and our results support these findings. Finally, we describe an example of a putative novel protein-coding gene that belongs to the ATP-binding cassette transporter gene family. The gene appeared to be present in chimpanzee and several other primates but part of the DNA sequence was absent from the human genome, thus suggesting that the gene is not functional in human. The novel exons, 3' UTRs and the putative gene described here are not present in the current annotations of chimpanzee genes and thus clearly show the weakness of existing homocentric gene catalogues. Taken together, our results point to a great transcriptional diversity in chimpanzee that has not been previously characterized.

## Conclusions

We have sequenced the chimpanzee transcriptome in frontal cortex and liver and provided a comprehensive catalogue of expressed RefSeq genes and numerous novel TRs. The vast majority of novel TRs was found within or in the vicinity of known genes and thus extends existing gene models, mainly by adding new exons and 3' UTRs. Furthermore, we have provided evidence of a gene that appears to have been lost in the human lineage. Our analyses highlight the great potential of combining RNA-Seq with splice junction predictions in order to generate a more complete understanding of transcriptome diversity.

## Materials and methods

### Sample preparation

Tissue samples from liver and frontal cortex were obtained through autopsies of two young chimpanzees (one female and one male), from the Kolmården Zoo, Sweden. The deep frozen tissues were cryo-sectioned and the slices recovered in QIAzol lysis buffer (Qiagen, Valencia, CA, USA). Ten 10-μm slices were used for the liver samples and 15 slices for frontal cortex. The miRNeasy (Qiagen) protocol for small tissue samples was used to prepare total RNA, which was subsequently treated with DNAse (Qiagen) to remove possible contamination from genomic DNA and further purified using the mini-elute kit (Qiagen). First strand cDNA synthesis was performed with the SuperSMART PCR cDNA synthesis kit (Clontech, Mountain View, CA, USA), using the protocol supplied by the manufacturer. The method enriches for full-length cDNAs by using specific oligomers for priming. A poly(A)-specific primer initiates the first strand synthesis of cDNA, thereby selecting for polyadenylated RNA while simultaneously keeping the concentration of ribosomal RNA low. The resulting single-stranded cDNA was amplified with the Advantage2 PCR kit (Clontech) using 27 amplification cycles. The amplified cDNA was cleaved with Rsa1 (NewEngland BioLabs, Ipswich, MA, USA) to partially remove the added oligonucleotide and 3' primer. The short cleavage products were eliminated in the size selection step in the library preparation preceding sequencing.

### Sequencing and mapping of reads

The cDNA was used to prepare a fragment library from each sample, according to the protocol supplied by the manufacturer (Applied Biosystems, Carlsbad, CA, USA) and sequenced using the SOLiD platform (Applied Biosystems). First, all four libraries were sequenced with a read length of 35 bp, brainF and brainM on a whole slide each, and liverF and liverM on half a slide each. Second, the libraries for brainF and liverF were sequenced with a read length of 50 bp, using a quarter of a slide per library. Sequencing reads have been deposited in the European Nucleotide Archive at EMBL with the accession number [EMBL: ERA000160].

Reads were aligned using the 'anchor-extend' method in the whole transcriptome analysis tool from Applied Biosystems [43]. In the alignment each read was split into two parts, or 'anchors' of length 25, which were then mapped to the chimpanzee March 2006 (panTro2) reference sequence. Each anchor that mapped uniquely to the reference was then extended as long as it was still matching the reference. This 'anchor-extend' approach makes it possible to map reads that partly overlap with a splice junction.

When computing the coverage signal, all reads that mapped to the exact same position and on the same strand were merged and only counted once. In this way we reduced the experimental bias caused by uneven PCR amplification of transcripts. The brainF and liverF libraries were sequenced twice with different read length (35 bp and 50 bp). Since there was a very high correlation between the replicates with 35 and 50 bp (Figure 2a,b), they were merged into one dataset per tissue.

### Quantification of expression levels of RefSeq genes

For each of the samples brainM, brainF, liverM and liverF, the coverage (that is, number of overlapping reads) was calculated for every position of the reference genome. To annotate genes, we used the 'Chimpanzee and human RefSeq genes' track in the UCSC Genome Browser [29,30]. This track defines genes based on the alignment of RefSeq [28] mRNAs. Thus, different isoforms from the same genomic locus will be annotated as different genes although they have the same common gene name (HGNC symbols).

To quantify expression levels, we calculated the average dcpm value [16] in the last 500 bp of the last exon. The average dcpm value was based on the whole last exon if it was shorter than 500 bp. In this way, each RefSeq gene was assigned a unique expression value. In order to establish a cut-off for expression of genes, we used the following strategy. We compared the expression levels, calculated as above, with the average dcpm values of randomly sampled regions outside of RefSeq exons. For each RefSeq gene, we sampled one random non-exonic region of the same length as was used for calculating the gene expression, that is, at most 500 bp. The sampling was done from the same chromosome as the gene was localized on and in this way we could compute average dcpm values for the same number of randomly sampled regions as the number of RefSeq genes. Assuming that most of the genome is transcribed only at very low levels [40], we then applied a cut-off so that 95% of the genes/TRs above the threshold were detected as transcribed and only 5% of the random regions. This is the same as having a 5% false discovery rate. However, we expect that some of the randomly sampled regions may represent biological transcripts, rather than randomly mapped reads, and thus the true false discovery rate is lower than 5%. Since the expression levels differed between the samples, the procedure was repeated once for each dataset, resulting in different cut-offs. After having established a cut-off for each of the samples, we used this to scan for novel TRs. All regions with a dcpm signal above the threshold over a stretch of at least 50 consecutive bases were considered to be transcribed, but to be included in the dataset of TRs we further required detection in the same tissue in both individuals.

### *De novo *discovery and annotation of transcribed regions

The expression threshold described above was subsequently used to define TRs across the entire genome. The complete set of TRs was compared to the RefSeq, mRNA and EST tables from the UCSC Genome Browser [29,30], containing information about genes, mRNAs or ESTs detected either in chimpanzee or in human. We then selected the TRs that did not overlap with any of the RefSeq genes, mRNAs or ESTs and such TRs were considered to be 'novel'. To increase the confidence in these novel TRs, we required them to be > 50 bp in length and to be present (that is, overlap at least one base pair) in the same tissue of both individuals.

### Detection of novel regions with no support in the human genome

The coordinates for all novel TRs were translated from the chimpanzee genome (panTro2) to the human genome (hg19) using the liftOver application available in the UCSC Genome Browser [29,30]. Regions that did not translate were BLASTed [31] to the entire human genome (including chromosomes labeled as random and unknown) and we then filtered out the ones that did not give any match (with e-value < 0.001). For the evolutionary analysis we used the liftOver tool to further translate the novel TRs to the macaque genome (rheMac2).

### Gene Ontology analysis

The DAVID functional annotation tool [44] was used to perform Gene Ontology classification of various lists of RefSeq genes. We generally used all genes in the genome as a background, focused on the ontology of biological processes and considered a corrected (Benjamini) *P*-value < 0.01 to be significant.

### Splice junction detection

To detect splice junctions, we analyzed the reads using the version 1.3.2 of the SplitSeek algorithm [32], available from the SOLiD software development community [45]. Since the SplitSeek algorithm is not applicable for the shorter read lengths, we used only the 50-bp reads from the brainF and liverF samples in this analysis. The analysis procedure and parameters were the same as in our previous study on mouse RNA-Seq data [32]. We required each prediction to be supported by at least two uniquely mapping reads, and the two parts of the junction to be separated by at most 1 Mb. All SplitSeek predictions are included in Additional file 2, which can be uploaded and viewed in the UCSC Genome Browser together with all identified TRs in the chimpanzee samples.

### Experimental validation of novel transcribed regions

RNA samples extracted from the female brain and liver were used to regenerate cDNAs for RT-PCR, including reactions without RT enzyme. We used the SMARTer™ Pico PCR cDNA synthesis Kit from Clontech (which has replaced the SuperSMART PCR cDNA kit used for library preparation) following the manufacturers instructions. Briefly, 1 μg of DNAase treated total RNA was included in each cDNA synthesis reaction and incubated at 42°C for 90 minutes. The cDNAs were purified using the Nucleospin Extract II kit and recovered in a final volume of 160 μl. Then, 1 μl of each cDNA reaction was used in 25 μl PCR reactions using the Advantage 2 PCR kit. The primers used in amplifications are given in Table S4 in Additional file 1. The amplifications were initiated by 1 minute incubation at 95°C followed by 35 cycles at 95°C for 15 s, 57 to 60°C for 30 s and 68°C for 30 s. The 10 × Advantage PCR buffer SA was used for primer pairs associated with *MN1*. We separated 10 μl of the PCR reactions using 2 to 3% NuSieve agarose gels.

## Abbreviations

bp: base pair; dcpm: depth of coverage per million reads; EST: expressed sequence tag; LTR: long terminal repeat; ncRNA: non-coding RNA; RNA-Seq: RNA sequencing; RT-PCR: reverse transcriptase PCR; TR: transcribed region; UTR: untranslated region.

## Authors' contributions

AW, LC and UG designed the study. AW and LC performed experiments. AA performed mapping of reads and together with AW, LC, LF and UG analyzed the data. AA and AW drafted the paper. All authors read and approved the final manuscript.

## Supplementary Material

Additional file 1 **Supplementary figures, tables and material concerning the novel gene**. Click here for file

Additional file 2 **Custom tracks summarizing our results, which can be uploaded and viewed in the UCSC Genome Browser**. Click here for file

## References

[B1] Chen FC Li WH Genomic divergences between humans and other hominoids and the effective population size of the common ancestor of humans and chimpanzees. Am J Hum Genet 2001 68 444 456 10.1086/318206 11170892 PMC1235277

[B2] Mikkelsen TS Hillier LW Eichler EE Zody MC Jaffe DB Yang S Enard W Hellmann I Lindblad-Toh K Altheide TK Initial sequence of the chimpanzee genome and comparison with the human genome. Nature 2005 437 69 87 10.1038/nature04072 16136131

[B3] Khaitovich P Hellmann I Enard W Nowick K Leinweber M Franz H Weiss G Lachmann M Paabo S Parallel patterns of evolution in the genomes and transcriptomes of humans and chimpanzees. Science 2005 309 1850 1854 10.1126/science.1108296 16141373

[B4] Enard W Khaitovich P Klose J Zollner S Heissig F Giavalisco P Nieselt-Struwe K Muchmore E Varki A Ravid R Doxiadis GM Bontrop RE Paabo S Intra-and interspecific variation in primate gene expression patterns. Science 2002 296 340 343 10.1126/science.1068996 11951044

[B5] Caceres M Lachuer J Zapala MA Redmond JC Kudo L Geschwind DH Lockhart DJ Preuss TM Barlow C Elevated gene expression levels distinguish human from non-human primate brains. Proc Natl Acad Sci USA 2003 100 13030 13035 10.1073/pnas.2135499100 14557539 PMC240739

[B6] Uddin M Wildman DE Liu G Xu W Johnson RM Hof PR Kapatos G Grossman LI Goodman M Sister grouping of chimpanzees and humans as revealed by genome-wide phylogenetic analysis of brain gene expression profiles. Proc Natl Acad Sci USA 2004 101 2957 2962 10.1073/pnas.0308725100 14976249 PMC365727

[B7] Somel M Franz H Yan Z Lorenc A Guo S Giger T Kelso J Nickel B Dannemann M Bahn S Webster MJ Weickert CS Lachmann M Paabo S Khaitovich P Transcriptional neoteny in the human brain. Proc Natl Acad Sci USA 2009 106 5743 5748 10.1073/pnas.0900544106 19307592 PMC2659716

[B8] Karaman MW Houck ML Chemnick LG Nagpal S Chawannakul D Sudano D Pike BL Ho VV Ryder OA Hacia JG Comparative analysis of gene-expression patterns in human and African great ape cultured fibroblasts. Genome Res 2003 13 1619 1630 10.1101/gr.1289803 12840040 PMC403735

[B9] Khaitovich P Kelso J Franz H Visagie J Giger T Joerchel S Petzold E Green RE Lachmann M Paabo S Functionality of intergenic transcription: an evolutionary comparison. PLoS Genet 2006 2 e171 10.1371/journal.pgen.0020171 17040132 PMC1599769

[B10] Gilad Y Rifkin SA Bertone P Gerstein M White KP Multi-species microarrays reveal the effect of sequence divergence on gene expression profiles. Genome Res 2005 15 674 680 10.1101/gr.3335705 15867429 PMC1088295

[B11] Blekhman R Oshlack A Chabot AE Smyth GK Gilad Y Gene regulation in primates evolves under tissue-specific selection pressures. PLoS Genet 2008 4 e1000271 10.1371/journal.pgen.1000271 19023414 PMC2581600

[B12] Sakate R Osada N Hida M Sugano S Hayasaka I Shimohira N Yanagi S Suto Y Hashimoto K Hirai M Analysis of 5'-end sequences of chimpanzee cDNAs. Genome Res 2003 13 1022 1026 10.1101/gr.783103 12727913 PMC430928

[B13] Sakate R Suto Y Imanishi T Tanoue T Hida M Hayasaka I Kusuda J Gojobori T Hashimoto K Hirai M Mapping of chimpanzee full-length cDNAs onto the human genome unveils large potential divergence of the transcriptome. Gene 2007 399 1 10 10.1016/j.gene.2007.04.013 17574350

[B14] Nagalakshmi U Wang Z Waern K Shou C Raha D Gerstein M Snyder M The transcriptional landscape of the yeast genome defined by RNA sequencing. Science 2008 320 1344 1349 10.1126/science.1158441 18451266 PMC2951732

[B15] Wilhelm BT Marguerat S Watt S Schubert F Wood V Goodhead I Penkett CJ Rogers J Bahler J Dynamic repertoire of a eukaryotic transcriptome surveyed at single-nucleotide resolution. Nature 2008 453 1239 1243 10.1038/nature07002 18488015

[B16] Hillier LW Reinke V Green P Hirst M Marra MA Waterston RH Massively parallel sequencing of the polyadenylated transcriptome of *C. elegans*. Genome Res 2009 19 657 666 10.1101/gr.088112.108 19181841 PMC2665784

[B17] Mortazavi A Williams BA McCue K Schaeffer L Wold B Mapping and quantifying mammalian transcriptomes by RNA-Seq. Nat Methods 2008 5 621 628 10.1038/nmeth.1226 18516045 PMC13303166

[B18] Cloonan N Forrest AR Kolle G Gardiner BB Faulkner GJ Brown MK Taylor DF Steptoe AL Wani S Bethel G Robertson AJ Perkins AC Bruce SJ Lee CC Ranade SS Peckham HE Manning JM McKernan KJ Grimmond SM Stem cell transcriptome profiling via massive-scale mRNA sequencing. Nat Methods 2008 5 613 619 10.1038/nmeth.1223 18516046

[B19] Sultan M Schulz MH Richard H Magen A Klingenhoff A Scherf M Seifert M Borodina T Soldatov A Parkhomchuk D Schmidt D O'Keeffe S Haas S Vingron M Lehrach H Yaspo ML A global view of gene activity and alternative splicing by deep sequencing of the human transcriptome. Science 2008 321 956 960 10.1126/science.1160342 18599741

[B20] Wang ET Sandberg R Luo S Khrebtukova I Zhang L Mayr C Kingsmore SF Schroth GP Burge CB Alternative isoform regulation in human tissue transcriptomes. Nature 2008 456 470 476 10.1038/nature07509 18978772 PMC2593745

[B21] Blekhman R Marioni JC Zumbo P Stephens M Gilad Y Sex-specific and lineage-specific alternative splicing in primates. Genome Res 2009 20 180 189 10.1101/gr.099226.109 20009012 PMC2813474

[B22] Babbitt C Fedrigo O Pfefferle A Boyle A Horvatth J Furey T Wray G Both noncoding and protein-coding RNAs contribute to gene expression evolution in the primate brain. Genome Biol Evol 2010 2010 67 79 10.1093/gbe/evq002 20333225 PMC2839352

[B23] t Hoen PA Ariyurek Y Thygesen HH Vreugdenhil E Vossen RH de Menezes RX Boer JM van Ommen GJ den Dunnen JT Deep sequencing-based expression analysis shows major advances in robustness, resolution and inter-lab portability over five microarray platforms. Nucleic Acids Res 2008 36 e141 10.1093/nar/gkn705 18927111 PMC2588528

[B24] Morrissy AS Morin RD Delaney A Zeng T McDonald H Jones S Zhao Y Hirst M Marra MA Next-generation tag sequencing for cancer gene expression profiling. Genome Res 2009 19 1825 1835 10.1101/gr.094482.109 19541910 PMC2765282

[B25] Brent MR How does eukaryotic gene prediction work?. Nat Biotechnol 2007 25 883 885 10.1038/nbt0807-883 17687368

[B26] Pickrell JK Marioni JC Pai AA Degner JF Engelhardt BE Nkadori E Veyrieras JB Stephens M Gilad Y Pritchard JK Understanding mechanisms underlying human gene expression variation with RNA sequencing. Nature 2010 464 768 772 10.1038/nature08872 20220758 PMC3089435

[B27] Bainbridge MN Warren RL Hirst M Romanuik T Zeng T Go A Delaney A Griffith M Hickenbotham M Magrini V Mardis ER Sadar MD Siddiqui AS Marra MA Jones SJ Analysis of the prostate cancer cell line LNCaP transcriptome using a sequencing-by-synthesis approach. BMC Genomics 2006 7 246 10.1186/1471-2164-7-246 17010196 PMC1592491

[B28] Pruitt KD Tatusova T Maglott DR NCBI reference sequences (RefSeq): a curated non-redundant sequence database of genomes, transcripts and proteins. Nucleic Acids Res 2007 35 D61 65 10.1093/nar/gkl842 17130148 PMC1716718

[B29] Rhead B Karolchik D Kuhn RM Hinrichs AS Zweig AS Fujita PA Diekhans M Smith KE Rosenbloom KR Raney BJ Pohl A Pheasant M Meyer LR Learned K Hsu F Hillman-Jackson J Harte RA Giardine B Dreszer TR Clawson H Barber GP Haussler D Kent WJ The UCSC Genome Browser database: update 2010. Nucleic Acids Res 2010 38 D613 619 10.1093/nar/gkp939 19906737 PMC2808870

[B30] The UCSC Genome Browser http://genome.ucsc.edu/

[B31] Altschul SF Gish W Miller W Myers EW Lipman DJ Basic local alignment search tool. J Mol Biol 1990 215 403 410 2231712 10.1016/S0022-2836(05)80360-2

[B32] Ameur A Wetterbom A Feuk L Gyllensten U Global and unbiased detection of splice junctions from RNA-seq data. Genome Biol 11 R34 10.1186/gb-2010-11-3-r34 20236510 PMC2864574

[B33] Kapranov P Cheng J Dike S Nix DA Duttagupta R Willingham AT Stadler PF Hertel J Hackermuller J Hofacker IL Bell I Cheung E Drenkow J Dumais E Patel S Helt G Ganesh M Ghosh S Piccolboni A Sementchenko V Tammana H Gingeras TR RNA maps reveal new RNA classes and a possible function for pervasive transcription. Science 2007 316 1484 1488 10.1126/science.1138341 17510325

[B34] Birney E Stamatoyannopoulos JA Dutta A Guigo R Gingeras TR Margulies EH Weng Z Snyder M Dermitzakis ET Thurman RE Kuehn MS Taylor CM Neph S Koch CM Asthana S Malhotra A Adzhubei I Greenbaum JA Andrews RM Flicek P Boyle PJ Cao H Carter NP Clelland GK Davis S Day N Dhami P Dillon SC Dorschner MO Fiegler H Identification and analysis of functional elements in 1% of the human genome by the ENCODE pilot project. Nature 2007 447 799 816 10.1038/nature05874 17571346 PMC2212820

[B35] Cheng J Kapranov P Drenkow J Dike S Brubaker S Patel S Long J Stern D Tammana H Helt G Sementchenko V Piccolboni A Bekiranov S Bailey DK Ganesh M Ghosh S Bell I Gerhard DS Gingeras TR Transcriptional maps of 10 human chromosomes at 5-nucleotide resolution. Science 2005 308 1149 1154 10.1126/science.1108625 15790807

[B36] Carninci P Kasukawa T Katayama S Gough J Frith MC Maeda N Oyama R Ravasi T Lenhard B Wells C Kodzius R Shimokawa K Bajic VB Brenner SE Batalov S Forrest AR Zavolan M Davis MJ Wilming LG Aidinis V Allen JE Ambesi-Impiombato A Apweiler R Aturaliya RN Bailey TL Bansal M Baxter L Beisel KW Bersano T Bono H The transcriptional landscape of the mammalian genome. Science 2005 309 1559 1563 10.1126/science.1112014 16141072

[B37] Gross SS Brent MR Using multiple alignments to improve gene prediction. J Comput Biol 2006 13 379 393 10.1089/cmb.2006.13.379 16597247

[B38] Benson DA Karsch-Mizrachi I Lipman DJ Ostell J Sayers EW GenBank. Nucleic Acids Res 2010 38 D46 51 10.1093/nar/gkp1024 19910366 PMC2808980

[B39] GenBank http://www.ncbi.nlm.nih.gov/genbank/

[B40] van Bakel H Nislow C Blencowe BJ Hughes TR Most "dark matter" transcripts are associated with known genes. PLoS Biol 2010 8 e1000371 10.1371/journal.pbio.1000371 20502517 PMC2872640

[B41] Harrow J Denoeud F Frankish A Reymond A Chen CK Chrast J Lagarde J Gilbert JG Storey R Swarbreck D Rossier C Ucla C Hubbard T Antonarakis SE Guigo R GENCODE: producing a reference annotation for ENCODE. Genome Biol 2006 7 Suppl 1 S4.1 S4.9 16925838 10.1186/gb-2006-7-s1-s4 PMC1810553

[B42] Cheng Z Ventura M She X Khaitovich P Graves T Osoegawa K Church D DeJong P Wilson RK Paabo S Rocchi M Eichler EE A genome-wide comparison of recent chimpanzee and human segmental duplications. Nature 2005 437 88 93 10.1038/nature04000 16136132

[B43] Ast G The alternative genome. Sci Am 2005 292 40 47 10.1038/scientificamerican0405-58 15915813

[B44] Huang da W Sherman BT Lempicki RA Systematic and integrative analysis of large gene lists using DAVID bioinformatics resources. Nat Protoc 2009 4 44 57 10.1038/nprot.2008.211 19131956

[B45] SplitSeek http://solidsoftwaretools.com/gf/project/splitseek/

