# Supplementary material for: Identification of novel exons and transcribed regions by chimpanzee transcriptome sequencing
Source: Genome Biol. 2010 Jul 23;11(7):R78. doi: 10.1186/gb-2010-11-7-r78 (PMC2926789; doi:10.1186/gb-2010-11-7-r78)
Supplement: Additional file 1 — Supplementary figures, tables and material concerning the novel gene. [file gb-2010-11-7-r78-S1.DOC]

# Supplementary Material

## Supplementary Figures and Figure legends

**
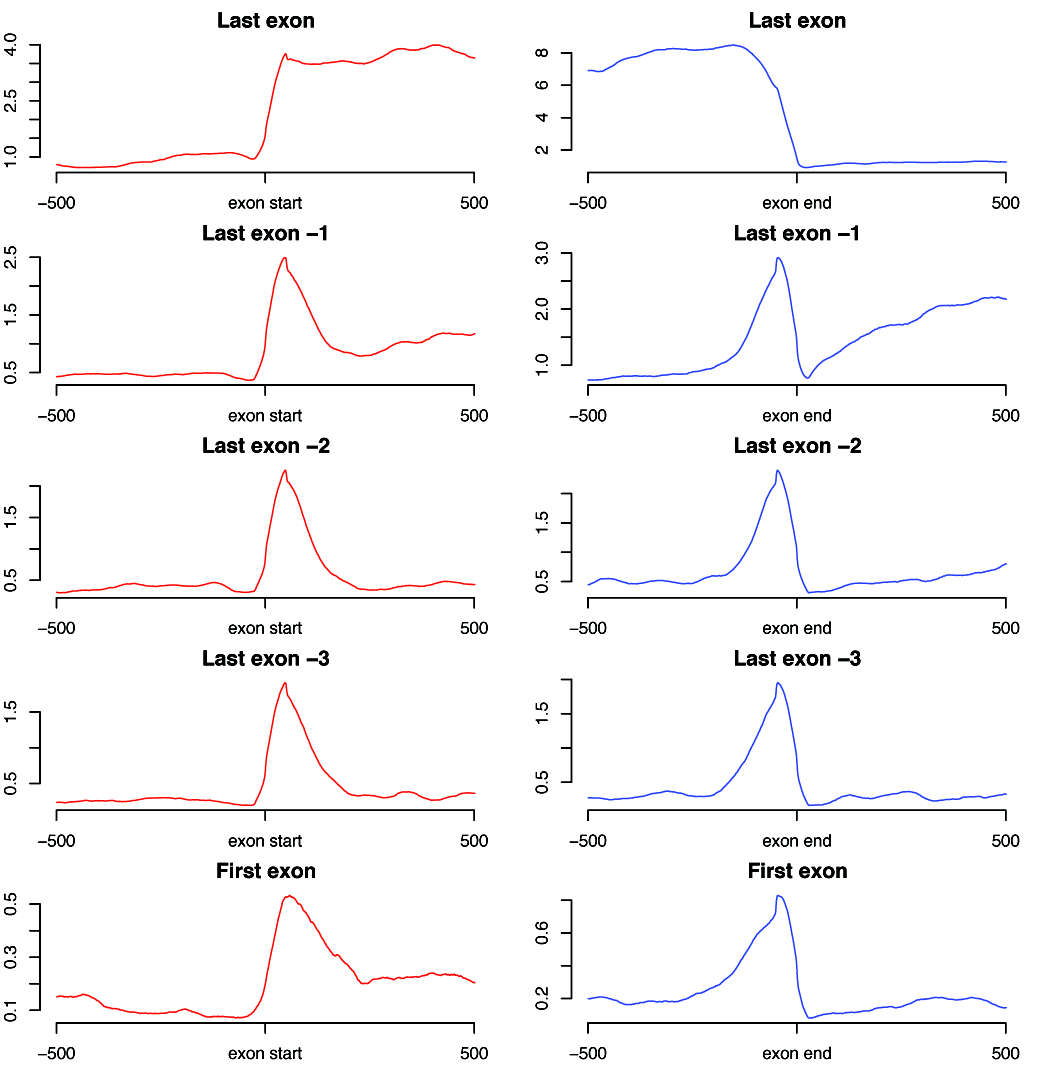
**

**Figure S1. Coverage signal for different exons in RefSeq genes.**

The figure displays the average coverage signal (y-axes) of exons for RefSeq genes (brainF sample). The top row shows the coverage of the last exons (in 3’ direction) of the RefSeq genes. The top-left figure shows the average coverage (red line) in a 500 bp window around the starts of the last exons. In the top-right figure, the coverage signal (blue line) is instead plotted in a 500 bp window surrounding the exon ends. The second row displays the coverage over the second last exon (Last exon-1), the third line shows Last exon-2, and so on. At the bottom is the average coverage over the first exons (5’ ends) of the RefSeq transcripts. The average coverage is highest at the 3’ends and then there is a clear decreasing trend towards the 5’ ends.

**
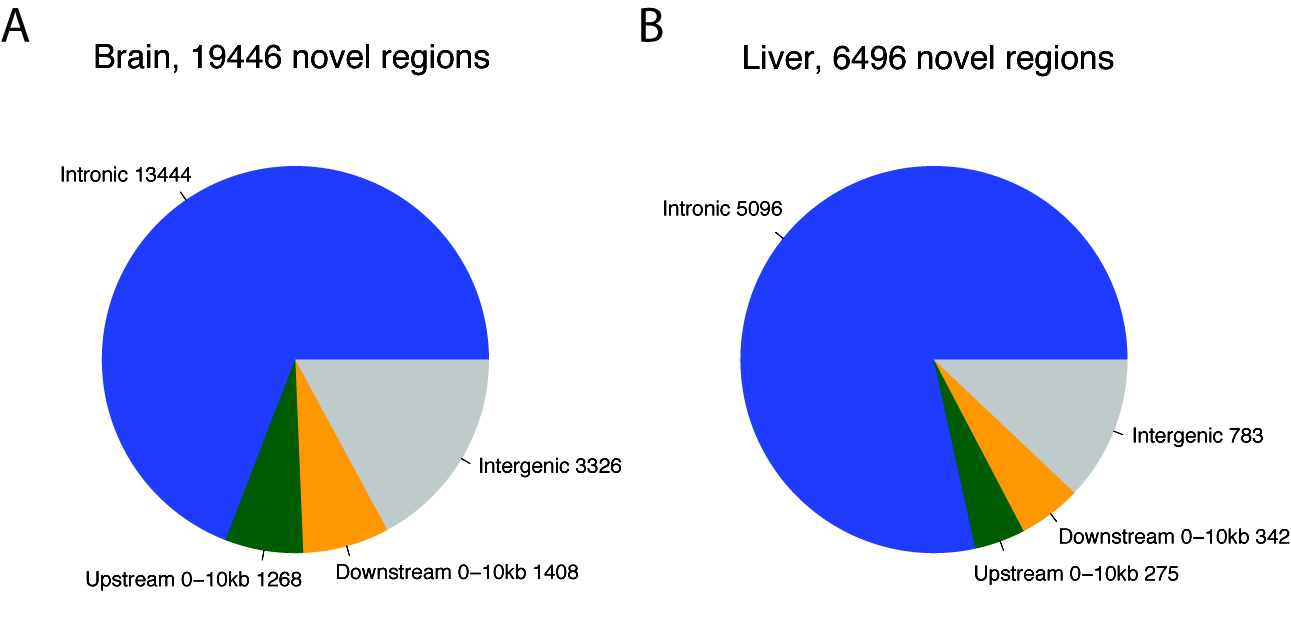
**

**Figure S2. Genomic distribution of novel transcribed regions (TRs).**

Illustration of the proportion of novel TRs located in different genomic locations: intronic in RefSeq genes, 0-10 kb upstream of RefSeq genes, 0-10 kb downstream of RefSeq genes and intergenically. Panel (A) dispays the distribution in brain samples and panel (B) in liver samples.


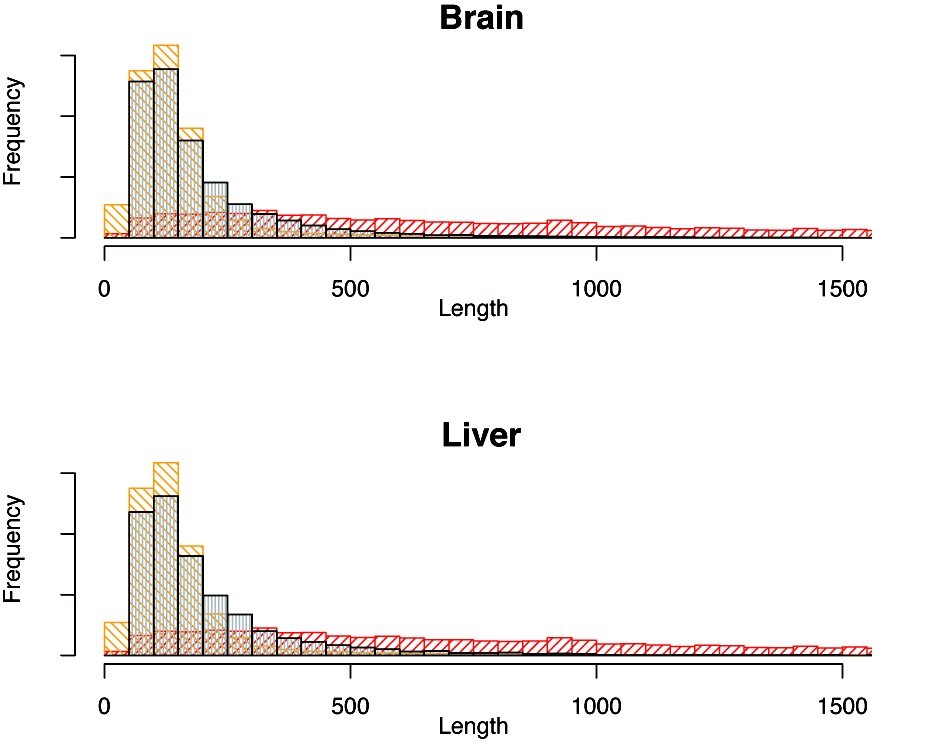


Figure S3. Length distributions for novel transcribed regions (TRs).

Brain is displayed in the upper panel and liver in the lower panel. The x-axis gives the length in basepairs and the y-axis denotes the frequency of novel transcribed regions with a certain length (grey). For comparison the frequency of Refseq exons of different lengths is shown, separating terminal exons (red) from all the aggregated group of ‘all other’ exons (yellow). We examined the length of novel TRs and found it to vary between 50 bp to 2,500 bp, with a median of 142 bp for brain and 151 bp for liver Terminal exons including 3’ UTRs were considerably longer (median = 864 bp) than ‘all other’ exons (median = 126 bp). The majority of novel TRs were found in the interval 50-200 bp and this coincided with a similar peak in the length distribution for ‘all other’ exons.

**A note on Figure S4-S7.** Since all four figures have the same layout the main themes are covered in a common note here. Panel (A) is a view from the UCSC Genome Browser with the RefSeq gene model at the top, followed by custom tracks that illustrate predicted splice junctions and expressed TRs. The lines at the bottom of panel (A) display the position of primers for validation and the experimentally validated junctions. Panel (B) displays gel pictures from the RT-PCR. The RT-PCR pruducts were separated on a 2 % agarose gel and a molecular marker (100 bp ladder from Invitrogen) was used for sizing the fragments. The ladder has 100 bp-intervalls, with a stronger band at 600 bp. The following four lanes on the gel represent RT-PCR of samples from brain and liver (female), the reactions were run both with, and without, reverse transcriptase. Where possible we have designed primers to amplify a fragment from the known transcript, to act as a control that the gene is transcribed. This was also used as a comparison to deduce if novel TRs were expressed at the same level, or lower, than the main transcript.


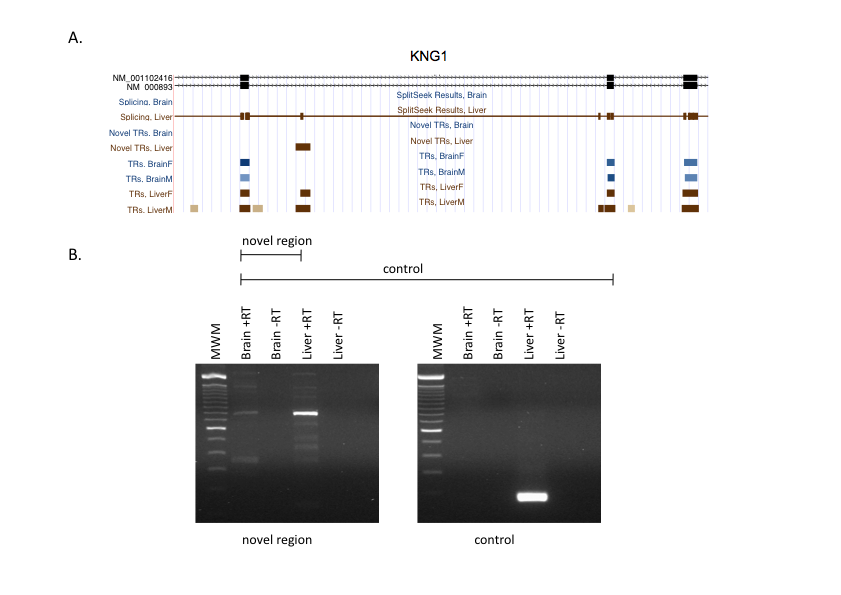


**Figure S4. *KNG1* is an example of a gene with a putative novel exon.**

The expected band, corresponding to the fragment called ’novel region’, was just above 200 bp. In the left gel in panel (B) there was a weak band for both the brain and the liver samples. This band represent the scenario where the two TRs are joined as in the schematic drawing in panel (A).There was also an extra band at 800 bp (stronger in liver than in brain) that represented the scenario where the intronic region between the two TRs was retained. Based on the results we concluded that there were two fragments, one from a spliced transcript and another from an unspliced transcript.

**
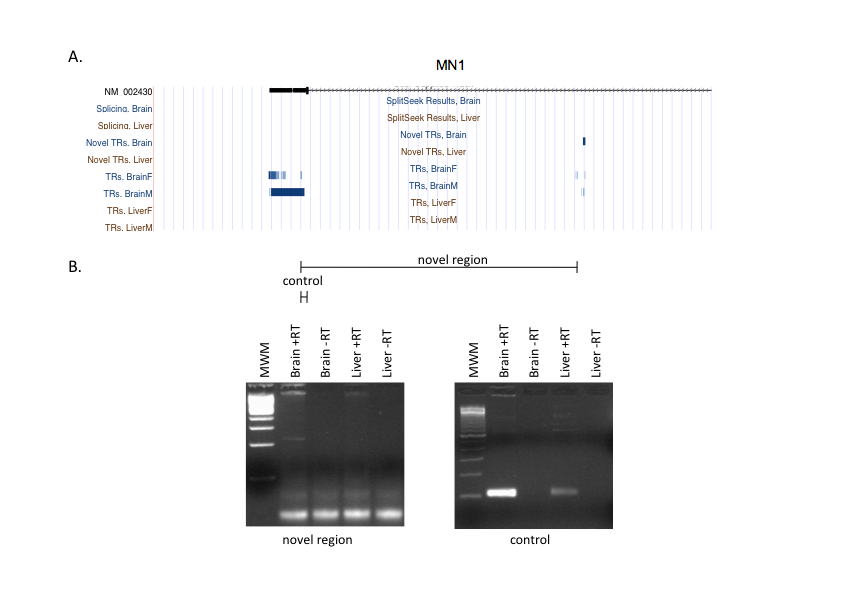
**

**Figure S5. *MN1* is an example of a gene with a putative novel exon.**

The positive control represents the known 3’ UTR and as seen in panel (B) the UTR is expressed in both tissues, although stronger in brain. The novel TR located upstream of the 3’ UTR was only validated in brain and the expression appeared to be lower than for the control. Thus it is possible that the novel exon is not constitutively included in all transcripts.

**
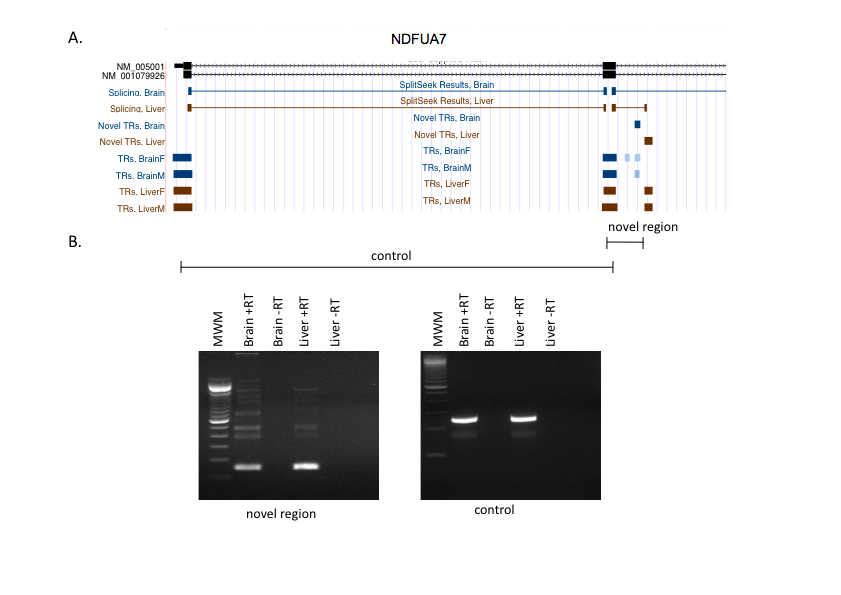
**

**Figure S6. *NDUFA7* is an example of a gene with a putative novel exon.**

The positive control connects the last exon with the 3’ UTR and this fragment was equally expressed in both tissues. The novel TR is located upstream of the last annotated exon and this putative exon was also expressed in both tissues, although more strongly in liver. There are several longer and weaker band on the gel and this probably reflects unspliced RNAs where the intron between the known and novel exons has been retained.


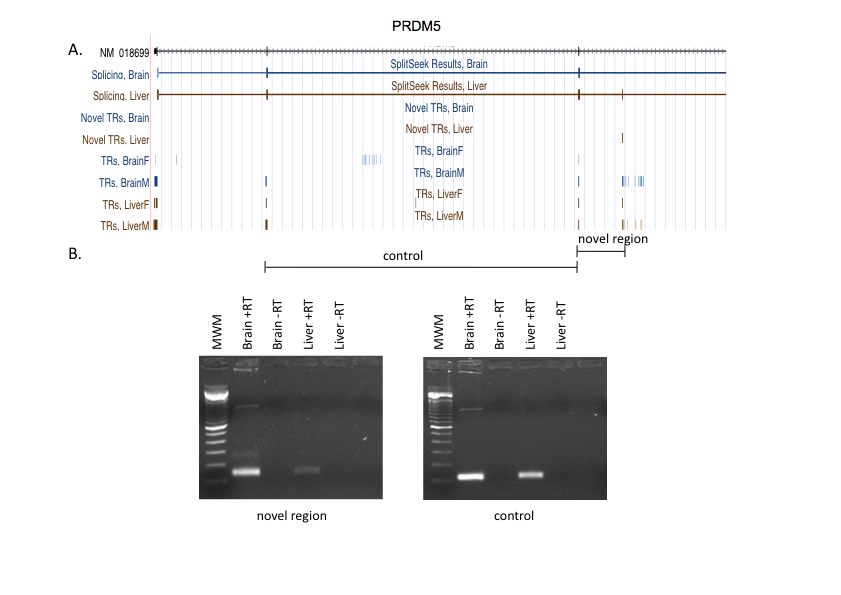


**Figure S7. *PRDM* is an example of a gene with a putative novel exon.**

The positive control represents splicing between two annotated exons and the resulting sequence fragment was detected in both tissues. The novel TR is a putative exon and the sequence fragment represents splcing between the novelk exon and one of the annotated exons. The novel TR is also detected in both tissues, although the expression is stronger in brain. The difference in expression levels is more pronounced for the novel TR than for the positive control region.

## Supplementary Tables

**Supplementary table S1:** Total number of nucleotides covered by transcribed regions (TRs) and the percentage of nucleotides classified as different genomic regions.

| **Sample** | **% of bp in last exon** | **% of bp in other exons** | **% of bp in intronic** | **% of bp in upstream** | **% of bp in downstream** | **% of bp in intragenic** | **Total number of bp in each sample** |
| --- | --- | --- | --- | --- | --- | --- | --- |
| ***BrainF*** | 23,6 | 11,8 | 43,1 | 4,9 | 7,9 | 8,7 | 25,566,540 |
| ***BrainM*** | 29,3 | 8,4 | 38,6 | 5,5 | 9,3 | 8,9 | 23,655,544 |
| ***LiverF*** | 26,9 | 15,5 | 40,6 | 4,1 | 6,4 | 6,5 | 20,329,733 |
| ***LiverM*** | 38,2 | 15,5 | 30,8 | 3,6 | 7,4 | 4,5 | 10,302,240 |

**Supplementary Table S2:** Overrepresented GO-categories for RefSeq genes with expression in both chimpanzee samples.

| **Top 10 overrepresented GO-categories** | **P-value (Benjamini)** |
| --- | --- |
| ***Brain only*** |  |
| nervous system development | 8.5E-38 |
| multicellular organismal development | 3.5E-18 |
| system development | 3.1E-16 |
| homophilic cell adhesion | 5.3E-15 |
| central nervous system development | 4.7E-14 |
| anatomical structure development | 3.9E-13 |
| cell-cell adhesion | 4.2E-12 |
| biological adhesion | 1.0E-10 |
| cell adhesion | 1.0E-10 |
| developmental process | 1.4E-10 |
| ***Liver* *only*** |  |
| response to external stimulus | 8.6E-19 |
| acute inflammatory response | 1.2E-18 |
| organic acid metabolic process | 1.9E-18 |
| response to wounding | 1.3E-17 |
| carboxylic acid metabolic process | 1.4E-17 |
| lipid metabolic process | 1.7E-11 |
| monocarboxylic acid metabolic process | 3.0E-11 |
| inflammatory response | 7.1E-11 |
| activation of plasma proteins during acute inflammatory response | 9.2E-11 |
| complement activation | 9.2E-11 |
| ***Brain and liver*** |  |
| primary metabolic process | 6.9E-60 |
| cellular metabolic process | 5.4E-59 |
| metabolic process | 5.8E-59 |
| macromolecule metabolic process | 1.2E-50 |
| cellular component organization and biogenesis | 1.2E-42 |
| macromolecule localization | 7.7E-38 |
| biopolymer metabolic process | 5.0E-37 |
| RNA processing | 5.2E-35 |
| mRNA metabolic process | 1.5E-34 |
| protein localization | 4.7E-34 |

**Supplementary Table S3:** Overrepresented GO-categories for RefSeq genes (including 10 bp upstream and downstream) with novel transcribed regions. Categories marked with ‘*’ were significant (p-value < 0.01) in the GO-analysis of expressed genes in the same tissue (Supplementary Table S1).

| **Top 10 overrepresented GO-categories** | **P-value (Benjamini)** |
| --- | --- |
| ***Brain only*** |  |
| cellular process* | 1.2E-17 |
| cellular macromolecule metabolic process | 1.9E-10 |
| macromolecule metabolic process | 1.6E-6 |
| cellular metabolic process | 1.8E-5 |
| nucleobase, nucleoside, nucleotide and nucleic acid metabolic process | 2.6E-5 |
| nervous system development* | 6.4E-5 |
| cellular nitrogen compound metabolic process | 8.5E-5 |
| nitrogen compound metabolic process | 1.0E-4 |
| transmission of nerve impulse* | 2.2E-4 |
| synaptic transmission* | 2.2E-4 |
| ***Liver only*** |  |
| oxoacid metabolic process | 4.6E-7 |
| carboxylic acid metabolic process* | 4.6E-7 |
| organic acid metabolic process* | 3.1E-7 |
| cellular ketone metabolic process | 3.4E-7 |
| response to external stimulus* | 3.8E-5 |
| regulation of response to stimulus | 6.2E-5 |
| cellular amine metabolic process | 8.5E-5 |
| response to chemical stimulus* | 8.1E-5 |
| protein-lipid complex assembly | 1.0E-4 |
| plasma lipoprotein particle assembly | 1.0E-4 |
| ***Brain and liver*** |  |
| cellular macromolecule metabolic process* | 6.3E-16 |
| cellular process* | 6.9E-16 |
| cellular metabolic process* | 1.2E-15 |
| cellular protein metabolic process* | 2.1E-13 |
| cellular macromolecule catabolic process* | 1.2E-10 |
| primary metabolic process* | 1.3E-10 |
| cellular protein catabolic process* | 1.3E-10 |
| post-translational protein modification* | 1.3E-10 |
| proteolysis involved in cellular protein catabolic process* | 1.3E-10 |
| macromolecule metabolic process* | 1.6E-10 |

**Supplementary table S4:** primers used for experimental validation of novel TRs.

| **Genomic region** | **Primers** |
| --- | --- |
| *NDUA7* | R1BL1:CGACGACATGATGATGGAAG  1ex1: TTAGGGGAAAAAGCCACTCA  2ex1: CCAAGGAGGCAAAGTAGTCG  R1AR1: GCCACAAGCTCTCCAACAAT |
| *UROS* | 3ex2: AATGCATTCCTGCAGCTTTT  5ex2: GCGTTAATACAACTTCCCTGTG |
| *KNG1* | R3AL1: TGGTGGCTGGATTGAACTTT  R3AR1: ACACCATATGGGCATTGAGC |
| *PRDM* | rR4BL1: CGAATGTGCATCTTCAGTCC  1ex4: ttgaagagacagtgattttgtgaa  R4AR1: GCGTCCCTATCAATGTCCTTAC  2ex4 TTGCTGCACTCTGAGCACTT |
| *MN1* | 1ex12: TGGACATACAGCAGCATGAA  2ex12: TGAAGTTCAGGTGATAATCATTCC |
| Novel gene | R5AL1: CATTGATCCTATTTATCAGGTGTG  R5AR1: AGTTCCAGTGAATTCAACCATTTGT  R5BL1: CCTCAATTTGATGCCTTGCT  R5BR1: TTGTCACCATGTTCCAGAGC  R5CL1: TATTGCCCTAATGGGAAGGT  R5CR1: TGTTGCCAAACTTGCTCTTG  1ex5: AATGTGACGCCCTCTGTACC  2ex5: CGGATGGTGAGAACAACAAA  3ex5 : TGTTCCTTTGGCAATGTTTG  4ex5: CCAGCGACTGTCCTTGCTA  R5GL1: TAAGCCCCAGATTCAACTGTATG  R5GR1: TTATTGCAGAAGAAAACCCAGTC |
| Intergenic on chromosome 1 | 1ex14: ccttcctaagaaagtatccagagg  2ex14: ccaggattgtaaatgccaac  3ex14: ggttcagtgaataggataaaagcaa  R14R1: tgtcatttcacgtttcttcca |

## Supplementary material regarding the predicted novel gene

**Coding sequence and translated amino acid sequence.**

>Coding sequence

ATGAGTGCTGAAGAGCTACAGGAGTTCAAGAAACCCATGAAAAAGGTGAGGTCAAGAATTGGCTACTGTCCTCAATTTGATGCCTTGCTGGAATATATGACTGCTCAGGAAATAATGATTATGTATGCCAGAATATGGGGAGTCTCTGAGCCCCAGATTGGGCTGTATGTGAACAAATGGTTGAATTCACTGGAACTGGAGTCTCATGCTGCCAGGCTTATCAACACCTACAGTGAAGGAAACAAACGTAGGCTGAGTACTGCTATTGCCCTAATGGGAAGGTCTTCAGTCATCTTCCTGGATGAGCCATCAACTGGCATGGACCCAGTAGCCAGACGCCTGCTCTGGAACATGGTGACAAAGACACGTGAAAGTGGAAAAGCCATCGTTATGACCTCCCACAGTATGGAGGAATGTGACGCCCTCTGTACCAGTCTAGCCATGATGGTGCAGGGGAAGTTCACTTGCTTGGGCAGCCCTCAGCATCTCAAGAGCAAGTTTGGCAACATTTACATCCTGAAGGTCAAGGTCAAGACTGAAGATAAATTAGAGGATTTTAAATGTTATGTTGCAACAACATTTCCAGGTAGTGTCTTAAAACATGAAAATCAAGGGATCCTTAACTACTACATTCCTAGCAAGGACAGTCGCTGGGGAAAGGTGTTTGGCATTTTGGAGGAAGCTAAAGAGCAATTCGATTTAGAAGACTATTCTGTCAGTCAGATCACACTGGAACAAGTCTTCCTGACCTTTGCTAACCCAGAGAAAGCATCCAGTGATGATGAAAACGAGGAAATAATGATCATGTATGCCAGATTATGGGGAGTCCCTAAGCCCCAGATTCAACTGTATGTGAAGAAATGGTTGAATTCACTTGAACTGGAGCCTCATGCTGATGAGCTTATCAACACCTACAGGGCTTTCAGTGTGTTCTACGCAGAAACCAGTTCAAGGACTGGGTTTTCTTCTGCAATAAAATGCTGA

>Protein sequence, 328 amino acids

MSAEELQEFKKPMKKVRSRIGYCPQFDALLEYMTAQEIMIMYARIWGVSEPQIGLYVNKWLNSLELESHAARLINTYSEGNKRRLSTAIALMGRSSVIFLDEPSTGMDPVARRLLWNMVTKTRESGKAIVMTSHSMEECDALCTSLAMMVQGKFTCLGSPQHLKSKFGNIYILKVKVKTEDKLEDFKCYVATTFPGSVLKHENQGILNYYIPSKDSRWGKVFGILEEAKEQFDLEDYSVSQITLEQVFLTFANPEKASSDDENEEIMIMYARLWGVPKPQIQLYVKKWLNSLELEPHADELINTYRAFSVFYAETSSRTGFSSAIKC-

**Similarity with other proteins**

The amino acid sequence above was used to query the NCBI database (http://www.ncbi.nlm.nih.gov/) of RefSeq proteins, using BlastP with standard parameters. The best match was a mouse protein belonging to the family of ATP-binding cassette proteins. The alignment with our query is shown below.

[GENE ID: 320631 Abca15](http://www.ncbi.nlm.nih.gov/sites/entrez?db=gene&cmd=search&term=320631&RID=XJVUX2JX01N&log$=geneexplicitprot&blast_rank=1) | ATP-binding cassette, sub-family A (ABC1), member 15 [Mus musculus]

Score = 386 bits (992), Expect = 1e-105, Method: Compositional matrix adjust. Identities = 181/246 (73%), Positives = 216/246 (87%), Gaps = 0/246 (0%)

Query 11 KPMKKVRSRIGYCPQFDALLEYMTAQEIMIMYARIWGVSEPQIGLYVNKWLNSLELESHA 70

K + KVRS+IGYCPQFDALLEYMT EIMIMYARIWG+SE QI YV K+LNSL+LESHA

Sbjct 1422 KNIVKVRSKIGYCPQFDALLEYMTGWEIMIMYARIWGISEHQIQPYVKKYLNSLDLESHA 1481

Query 71 ARLINTYSEGNKRRLSTAIALMGRSSVIFLDEPSTGMDPVARRLLWNMVTKTRESGKAIV 130

LI+TYSEGNKRRLSTAIA MG+ SVIFLDEPSTGMDP ARRLLW+ V K RESGKAI+

Sbjct 1482 NSLISTYSEGNKRRLSTAIATMGKPSVIFLDEPSTGMDPRARRLLWDTVIKIRESGKAII 1541

Query 131 MTSHSMEECDALCTSLAMMVQGKFTCLGSPQHLKSKFGNIYILKVKVKTEDKLEDFKCYV 190

+TSHSMEEC+ALCT L++MV+G+ TCLGSPQ+LK+KFGNIYILK KVK+ + L++FK ++

Sbjct 1542 ITSHSMEECEALCTRLSIMVRGRLTCLGSPQYLKNKFGNIYILKAKVKSGETLDEFKNFI 1601

Query 191 ATTFPGSVLKHENQGILNYYIPSKDSRWGKVFGILEEAKEQFDLEDYSVSQITLEQVFLT 250

TFPGS L+ ENQGILNY IP K++ WGKVFGILE+AKEQ++LEDYS+SQITL+QVFL+

Sbjct 1602 TLTFPGSELQQENQGILNYCIPRKNNSWGKVFGILEKAKEQYNLEDYSISQITLDQVFLS 1661

Query 251 FANPEK 256

FA+ ++

Sbjct 1662 FADQDR 1667
